# Increased Trimethylamine N-Oxide Is Not Associated with Oxidative Stress Markers in Healthy Aged Women

**DOI:** 10.1155/2019/6247169

**Published:** 2019-09-16

**Authors:** Robert Antoni Olek, Joanna Jolanta Samulak, Angelika Katarzyna Sawicka, Dace Hartmane, Solveiga Grinberga, Osvalds Pugovics, Wieslawa Lysiak-Szydlowska

**Affiliations:** ^1^Department of Bioenergetics and Nutrition, Gdansk University Physical Education and Sport, Gorskiego 1, 80-336 Gdansk, Poland; ^2^Latvian Institute of Organic Synthesis, Riga, Latvia; ^3^Powislanski College, Department of Health Sciences, 82-500 Kwidzyn, Poland

## Abstract

Increased plasma trimethylamine N-oxide (TMAO) levels have been associated with cardiovascular diseases (CVD). L-carnitine induces TMAO elevation in human blood, and thus, it has been suggested as developing atherosclerosis. The aim of this study was to determine the relation between selected markers of oxidative stress and plasma TMAO concentration induced by L-carnitine supplementation for 24 weeks in healthy aged women. Twenty aged women were supplemented during 24 weeks with either 1500 mg L-carnitine-L-tartrate (*n* = 11) or isonitrogenous placebo (*n* = 9) per day. Fasting blood samples were taken from antecubital vein. L-carnitine supplementation induced an increase in TMAO, but not in *γ*-butyrobetaine (GBB). Moreover, there were no significant changes in serum ox-LDL, myeloperoxidase, protein carbonyls, homocysteine, and uric acid concentrations due to supplementation. Significant reduction in white blood cell counts has been observed following 24-week supplementation, but not attributable to L-carnitine. Our results in healthy aged women indicated no relation between TMAO and any determined marker of oxidative stress over the period of 24 weeks. At the same time, plasma GBB levels were not affected by L-carnitine supplementation. Further clinical studies of plasma GBB level as a prognostic marker are needed.

## 1. Introduction

Important role in initiation and progression of multiple cardiovascular diseases (CVD) such as atherosclerosis, hypertension, and coronary heart disease plays endothelial dysfunction [1]. Endothelial dysfunction has been strongly associated to reactive oxygen species (ROS) production and dysregulation of oxidant-antioxidant balance [2]. The well-defined mediator of endothelial dysfunction is oxidized LDL (ox-LDL), which leads to the formation of “foam cells” [3, 4]. The main oxidant responsible for LDL oxidation is hypochlorous acid, produced by the myeloperoxidase (MPO) [5–7]. Epidemiological studies have shown that higher serum MPO is recognized as both a risk factor for the development of coronary artery disease [8] and can be predictive of future cardiac events and outcome [9–11].

In recent years, the role of microbiome in the pathophysiology of CVD has gained significant interest. Intestinal microbiota metabolism and CVD have been linked through trimethylamine N-oxide (TMAO) [12]. Increased plasma TMAO levels have been associated with increased risk for major adverse cardiovascular events defined as death, myocardial infarction, or stroke [13–16]. TMAO may be produced by the intestinal microbiota from L-carnitine, via the microbiota-dependent intermediate metabolite *γ*-butyrobetaine (GBB) [17, 18]. Since dietary L-carnitine induces TMAO elevation in human blood [19–21], it has been suggested that L-carnitine increases atherosclerosis [12]. On the contrary, L-carnitine treatment has been demonstrated to attenuate the development of endothelial dysfunction in spontaneously hypertensive rats [22, 23] and many studies presented L-carnitine as an antioxidant effectively scavenging ROS in various *in vitro* and *in vivo* models [24].

The aim of the current study was to determine the association between selected markers of oxidative stress and plasma TMAO concentration induced by L-carnitine supplementation for 24 weeks in healthy aged women.

## 2. Materials and Methods

### 2.1. Subjects

The participants of the study were originally recruited to another study aimed at evaluating the effect of L-carnitine supplementation on skeletal muscle function [25] (individuals with CVD; liver and kidney diseases; gastrointestinal disorders, including stomach ulcers and erosions; neuromuscular disease; diabetes; and other severe chronic diseases were excluded during the recruitment process). The Independent Bioethics Commission for Research at Medical University of Gdansk has approved the study protocol (NKBBN/354-304/2015). Before starting the experimental procedure, all subjects gave their written informed consent. Twenty women in the age ranged from 65 to 70 years were supplemented during 24 weeks with either 1500 mg L-carnitine-L-tartrate (*n* = 11) or isonitrogenous placebo (*n* = 9) per day. The participants were examined 3 times throughout the period of the study, prior to the study, after 12 and 24 weeks of supplementation.

### 2.2. Blood Sampling

Blood samples were taken from antecubital vein. White blood cell (WBC) count and differential leukocyte count were determined using an automated hematology analyzer (Sysmex XT 2000, Global Medical Instrumentation, Inc.) in the whole blood. Plasma and serum were obtained by centrifugation at 2000 g at 4°C for 10 min. Aliquots were stored at -80°C for later analyses.

### 2.3. Biochemical Determination

Plasma TMAO and GBB were determined by the UPLC/MS/MS method as described previously [26]. Serum ox-LDL and MPO concentrations were determined by the enzyme immunoassay method using commercially available kits (ox-LDL—Immunodiagnostik AG, Bensheim, Germany; MPO—Abnova Corp., Taipei, Taiwan); protein carbonyls (PC), spectrophotometrically using Protein Carbonyl Colorimetric Assay Kit (Cayman Chemical, Michigan, USA); homocysteine (Hcy), by immunochemiluminescence method using Immulite 2000 XPi (Siemens Healthcare Diagnostics Inc.); and uric acid (UA), using standard automatic analyzer Cobas6000 (Roche Diagnostics, Mannheim, Germany).

### 2.4. Statistical Analyses

All calculations were performed using software Statistica 13.1 (Dell Inc., Tulsa, OK, USA). The analysis of variance (ANOVA) for repeated measurements was performed to examine the interaction between the treatment and time. In case the ANOVA yielded a significant effect, a Tukey-Kramer test was used for post hoc comparisons. A probability level *p* < 0.05 was considered statistically significant. All data are expressed as mean ± SE (standard error).

## 3. Results

**Figure 1:**
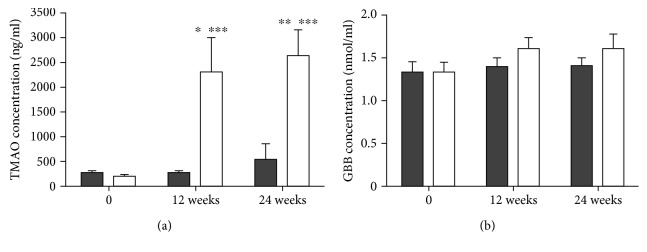
Plasma TMAO (a) and GBB (b) concentrations in *placebo* (grey bars) and in L-carnitine supplemented (white bars) groups. ^∗^*p* < 0.05 and ^∗∗^*p* < 0.01 as compared to *placebo* in the same time points, ^∗∗∗^*p* < 0.001 as compared to 0 time in the same group.

L-carnitine supplementation induced a tenfold increase in TMAO, observed in the midpoint of the study and maintained elevated until the end of the supplementation period (Figure 1(a)). At the same time points, plasma GBB in the supplemented group were not different (*p* > 0.05) compared to placebo (Figure 1(b)).

The data of all determined oxidative stress biomarkers are summarized in Table 1. There were no significant changes in PC, ox-LDL, MPO, UA, and Hcy serum concentrations due to 24-week supplementation.

Significant reduction in WBC, mostly in lymphocyte and monocyte counts, has been observed following 24-week supplementation, but not attributable to L-carnitine (Table 2). Despite significant decrease in lymphocyte counts, the mean values of the neutrophil-to-lymphocyte ratio (NLR) remained at the level ≤1.8 (Table 2).

## 4. Discussion

Similar to previously reported studies [19–21], L-carnitine supplementation induced increased plasma TMAO in humans. TMAO elevation was not related to any determined markers of oxidative stress nor WBC counts in aged women.

Since TMAO may directly act as an oxidant [27], animal treatment by TMAO in drinking water induces ROS generation [28, 29]. Moreover, inhibition of TMAO production in pathophysiological condition attenuates oxidative stress [30–32]. Thus, elevated circulating TMAO has been presented as a contributing factor in endothelial [30], cardiac [33], and renal [31] dysfunctions in animal models. Although we observed a 10-fold increase in plasma TMAO concentration of L-carnitine supplemented group, we could not measure TMAO-evoked changes in the oxidant/antioxidant status using serum PC or ox-LDL in the human subjects. Similarly, Fukami and colleagues [19] indicated that 6-month oral L-carnitine supplementation in hemodialysis patients significantly increased plasma TMAO, but markers of oxidative stress (malondialdehyde) and vascular injury (vascular cell adhesion molecule and intercellular adhesion molecule) decreased.

Despite a number of studies showing a positive correlation between elevated plasma TMAO concentration and an increased risk for major adverse cardiovascular events [13–16], higher plasma TMAO may be merely a marker of other cardiovascular risk factors, such as disturbed gut-blood barrier [34], high salt intake [35], or low glomerular filtration rates (GFR) [13]. GFR of the subjects participating in the current study were within the normal range [36], and four months after cessation of L-carnitine treatment, TMAO reached a level comparable to the values observed before supplementation started [36, 37]. Furthermore, recent study showed that chronic, low-dose TMAO may be beneficial for the circulatory system [38].

GBB, intermediate in gut microbial metabolism of L-carnitine, has also been considered as a proatherogenic factor [18, 39–41]. Increase in plasma GBB concentration was shown to be related to the development of atherosclerosis in apolipoprotein E knockout (ApoE-/-) mice [18, 40]. Moreover, higher circulating GBB has been presented in carotid atherosclerosis patients [39] and has been associated with increase in total atheroma volume after cardiac transplantation [41]. On the contrary, L-carnitine supplementation did not induce elevation in plasma GBB neither in our subjects nor in healthy pregnant women [42]. GBB is produced during L-carnitine biodegradation by *Enterobacteriaceae* such as *Escherichia coli* [43] and excreted primarily in feces [44]. Therefore, it seems plausible that disturbed gut-blood barrier may increase penetration of L-carnitine metabolites into the bloodstream [34], suggesting gut-blood barrier permeability as a diagnostic marker in CVD [45].

The pathogenesis of human CVD is linked to various ROS sources [2]. Some of the markers, associated with oxidative stress, have been proposed as clinical prognostic indicators for patients with CVD [9, 11, 46–51]. MPO promotes oxidative modification of proteins and lipids in CVD via various mechanisms [5]. According to EPIC-Norfolk Prospective Population Study [11], MPO level is associated with the risk of CVD in apparently healthy individuals. In the Aging and Longevity Study in the Sirente geographic area, the lowest all-cause mortality risk was observed in the group with plasma MPO ≤ 61.5 *μ*g/L [46]. Similarly, the level of circulating UA has been considered as a predictor of all-cause mortality in CVD patients [47]. Despite its important antioxidant effect, the mechanisms whereby UA promotes atherosclerosis are probably via UA-derived free radicals' generation [52]. As a substrate for MPO, UA is oxidized to the urate radical and then urate hydroperoxide [53], suggesting that UA may affect the progression of endothelial dysfunction [54]. Indeed, the risk for mortality increases markedly at UA serum levels > 7.5 mg/dL [~450 *μ*mol/L] [48]. Epidemiological studies have also investigated the relationship between CVD and Hcy levels in the blood [50, 51, 55]. Hcy impairs endothelial function by producing hydrogen peroxide [56] and superoxide anion [57] and can enhance LDL oxidation [58]. Still none of these markers have changed in the supplementation period, despite changes in the TMAO level, and stayed within the normal ranges.

L-carnitine has a protective effect on the oxidative-induced decrease in low-molecular-weight thiols and lipid peroxidation in plasma [59], and L-carnitine supplementation reduces ox-LDL in patients with diabetes [60]. However, neither ox-LDL nor PC concentrations were affected by L-carnitine supplementation in the present study. This may be due to the lack of oxidative stress, since ox-LDL [61, 62] and PC [63] values were similar to previously reported in healthy control subjects at corresponding ages.

Significant reduction in WBC, mostly in lymphocyte and monocyte counts, has been observed following 24-week supplementation, but not attributable to L-carnitine. Since the study protocol started in winter and finished in summer, it seems plausible that seasonal variations may be responsible for the variations in complete blood count [64, 65]. At the same time, NLR, an index of systemic inflammation associated with subclinical atherosclerosis [66], maintained at the level of ≤1.8, comparable to the control subjects [67]. NLR > 2.4 predicts with 80% probability of the carotid plaques, and NLR > 3.68 gives 97% probability [68]. Moreover, other inflammatory markers, i.e., vascular cell adhesion molecule, intercellular adhesion molecule, L-selectin, P-selectin, C-reactive protein, tumor necrosis factor *α*, and interleukin-6, were not affected by the L-carnitine supplementation [36].

## 5. Conclusion

Our results in healthy aged women indicated no relation between TMAO and any determined marker of oxidative stress over the period of 24 weeks. At the same time, plasma GBB levels were not affected by L-carnitine supplementation. Further clinical studies of plasma GBB level as a prognostic marker are needed.

## Figures and Tables

**Table 1:** Oxidative stress markers in L-carnitine and *placebo* groups: 0, 12, and 24 weeks of supplementation.

	L-carnitine			Placebo		
	0 week	12 weeks	24 weeks	0 week	12 weeks	24 weeks
PC (nmol·mg protein^−1^)	0.84 ± 0.04	0.82 ± 0.05	0.77 ± 0.04	0.77 ± 0.05	0.80 ± 0.04	0.84 ± 0.10
ox-LDL (*μ*g·L^−1^)	45 ± 14	60 ± 19	57 ± 18	40 ± 4	73 ± 17	44 ± 7
MPO (*μ*g·L^−1^)	60.9 ± 5.0	59.8 ± 6.7	55.9 ± 6.1	53.9 ± 3.9	48.1 ± 5.7	48.5 ± 4.8
UA (*μ*mol·L^−1^)	283 ± 15	307 ± 21	312 ± 24	289 ± 22	292 ± 31	306 ± 24
Hcy (*μ*mol·L^−1^)	11.8 ± 1.0	13.4 ± 1.8	13.1 ± 1.4	11.5 ± 1.1	11.9 ± 0.9	12.9 ± 1.4

PC: protein carbonyls; ox-LDL: oxidized low-density lipoprotein; MPO: myeloperoxidase; UA: uric acid; Hcy: homocysteine.

**Table 2:** Circulating white blood cell counts in L-carnitine and *placebo* groups: 0, 12, and 24 weeks of supplementation.

	L-carnitine			Placebo		
	0 week	12 weeks	24 weeks	0 week	12 weeks	24 weeks
Leuko (10^9^·L^−1^)^∗^	6.5 ± 0.5	6.0 ± 0.5	5.6 ± 0.4	6.3 ± 0.6	6.0 ± 0.7	5.8 ± 0.5
Neutro (10^9^·L^−1^)	3.1 ± 0.3	3.0 ± 0.4	2.9 ± 0.3	3.2 ± 0.3	3.2 ± 0.4	3.3 ± 0.3
Lympho (10^9^·L^−1^)^∗^	2.5 ± 0.3	2.1 ± 0.2	2.0 ± 0.2	2.4 ± 0.4	2.1 ± 0.3	1.9 ± 0.2
NLR	1.4 ± 0.2	1.5 ± 0.2	1.6 ± 0.2	1.5 ± 0.2	1.6 ± 0.2	1.8 ± 0.2
Mono (10^9^·L^−1^)^∗^	0.56 ± 0.03	0.55 ± 0.04	0.50 ± 0.03	0.53 ± 0.06	0.47 ± 0.05	0.48 ± 0.06
Platelets (10^9^·L^−1^)	285 ± 14	291 ± 14	282 ± 15	266 ± 18	268 ± 18	253 ± 18

^∗^ *p* < 0.05 main time effect. Leuko: leukocytes; Neutro: neutrophils; Lympho: lymphocytes; NLR: neutrophil-to-lymphocyte ratio (NLR); Mono: monocytes.

## Data Availability

The data used to support the findings of this study are available from the corresponding author upon request.
